# Phosphodiesterase Type 5 Inhibitors in Men With Erectile Dysfunction and the Risk of Alzheimer Disease: A Cohort Study

**DOI:** 10.1212/WNL.0000000000209131

**Published:** 2024-02-07

**Authors:** Matthew Adesuyan, Yogini H. Jani, Dana Alsugeir, Robert Howard, Chengsheng Ju, Li Wei, Ruth Brauer

**Affiliations:** From the Research Department of Practice and Policy (M.A., Y.H.J., D.A., C.J., L.W., R.B.), UCL School of Pharmacy; Centre for Medicines Optimisation Research and Education (M.A., Y.H.J.), University College London Hospitals NHS Foundation Trust, United Kingdom; Pharmacy Practice Department (D.A.), College of Clinical Pharmacy, Imam Abdulrahman Bin Faisal University, Dammam, Saudi Arabia; and Division of Psychiatry (R.H.), University College London, United Kingdom.

## Abstract

**Background and Objectives:**

Repurposing phosphodiesterase type 5 inhibitors (PDE5Is) as drugs for Alzheimer disease (AD) risk reduction has shown promise based on animal studies. However, evidence in humans remains inconclusive. Therefore, we conducted a cohort study to evaluate the association between PDE5I initiation compared with nonuse and the risk of developing AD in men with erectile dysfunction (ED).

**Methods:**

Using electronic health records from IQVIA Medical Research Data UK (formerly known as the THIN database), we identified men aged ≥40 years with a new diagnosis of ED between 2000 and 2017. Individuals with a previous diagnosis of dementia, cognitive impairment, confusion, or prescription for dementia symptoms were excluded. The occurrence of incident AD was identified using diagnostic read codes. To minimize immortal-time bias, PDE5I initiation was treated as a time-varying exposure variable. Potential confounders were adjusted using inverse probability of treatment weighting based on propensity scores. Cox proportional hazard models were used to estimate the adjusted hazard ratio (HR) with 95% CIs. A secondary analysis explored the association between AD and the cumulative number of PDE5I prescriptions. Sensitivity analyses included lag (delay) periods of 1 and 3 years after cohort entry to address the prodromal stage of AD.

**Results:**

The study included 269,725 men, with 1,119 newly diagnosed with AD during a median follow-up of 5.1 (interquartile range 2.9–8.9) years. The adjusted HR in PDE5I initiators compared with nonuse was 0.82 (95% CI 0.72–0.93). The associated risk of AD decreased in individuals issued >20 prescriptions: HR 0.56 (95% CI 0.43–0.73) for 21–50 prescriptions and HR 0.65 (95% CI 0.49–0.87) for >50 prescriptions. Sensitivity analysis with a 1-year lag period supported the primary findings (HR 0.82, 95% CI 0.72–0.94), but the results differed with the inclusion of a 3-year lag period (HR 0.93, 95% CI 0.80–1.08).

**Discussion:**

PDE5I initiation in men with ED was associated with a lower risk of AD, particularly in those most frequently issued prescriptions. The differences between primary and sensitivity analyses highlight the need to explore the optimal lag period. To enhance the generalizability of our findings, a randomized controlled trial including both sexes and exploring various PDE5I doses would be beneficial to confirm the association between PDE5I and AD.

## Introduction

Alzheimer disease (AD) is the most common form of dementia, accounting for approximately 50%–75% of cases in the United Kingdom and is a leading cause of death.^1,2^ All-cause dementia is estimated to affect 57 million people globally, with cases predicted to increase to 157 million by 2050.^3^ There is no cure for AD. However, monoclonal antibodies targeting the immune system have recently been developed to promote clearance of β-amyloid plaques in the brains of people living with early stage AD.^4,5^ These new treatments demonstrate positive progress in the identification of therapeutic agents to treat individuals with AD. Nonetheless, interventions that can prevent or delay the development of AD are also essential for promoting healthy aging and should continue to be an area of critical research.

Drug repurposing is the investigation of existing drugs for new uses and can be a cost-effective and timely method for identifying new therapeutic options.^6^ Phosphodiesterase type 5 inhibitors (PDE5Is) are one of the most widely used drugs that have been repurposed. Sildenafil, the first PDE5I, was originally developed for the treatment of hypertension and angina.^7^ The intended vasodilatory effects were also found to cause smooth muscle relaxation in the corpus cavernosum, making sildenafil (Viagra) an effective repurposed treatment option for erectile dysfunction (ED).^7^ The repurposing possibility of PDE5I continued beyond ED, and in 2005, sildenafil also became licensed for the treatment of pulmonary arterial hypertension (PAH).^8^

The primary clinical effects of PDE5I are a result of raised cyclic guanosine monophosphate (cGMP), a secondary messenger that is degraded by the phosphodiesterase enzyme (PDE).^9^ The relationship between the levels of cGMP and memory has been explored previously, with studies showing low levels of cGMP in tandem with raised levels of PDE in the brains of people with AD.^10,11^ These findings have led to several PDE5I studies in animal models, which have demonstrated possible neuroprotective benefits.^12^ However, evidence of neuroprotective effects in humans is not conclusive. A specific PDE5I, tadalafil, has been shown to improve cerebral blood flow, cognition, and neuroinflammation in men with ED.^13,14^ Contrary to these findings, a recent trial ending in 2018 found a nonsignificant increase in the cerebral blood flow after single use of tadalafil.^15^

Two recent cohort studies investigating the association between PDE5I and AD have been conducted in the United States. Work by Fang et al.^16^ reported that use of sildenafil in older adults was associated with a 69% reduced risk of AD compared with nonuse, whereas Desai et al.^17^ found no evidence of association between PDE5I and risk of AD when compared with endothelin receptor antagonists in people with PAH. Both studies produced conflicting results, but also have limited comparability because the study population, design, and comparator all differed. Fang et al. did not stipulate an underlying target population for their nonuser comparator study; therefore, a level of confounding by indication may be apparent in their reported effect estimate. Desai et al. carried out a new-user active comparator study. However, the number of individuals included in analyses was relatively small and a median follow-up of under 2 years may have rendered their study underpowered to detect differences of small magnitude in a rare outcome.

Given the discrepancy in findings between previous studies and the clinical data supporting the potential repurposing effects of PDE5I on neurologic disease, we conducted a large population-based cohort study to determine whether the use of PDE5I compared with nonuse is associated with a lower risk of AD in a homogenous population of men with ED.

## Methods

### Data Source

This study was conducted using IQVIA Medical Research Data (IMRD), incorporating data supplied by The Health Improvement Network (THIN) database, a propriety database of Cegedim SA. This database contains pseudonymized electronic primary care data from more than 16 million patients in the United Kingdom, representing approximately 6% of the population.^18^ The validity of the IMRD-UK database (previously known as THIN) for research has been demonstrated in several studies,^19-21^ including the generalizability and study-specific accuracy of dementia diagnoses.^22^ Data within the database includes demographic information, lifestyle (e.g., smoking and alcohol consumption), laboratory results, medical diagnoses, and prescribing information.

### Study Population

We conducted a population-based cohort study in men aged ≥40 years with a new diagnosis of ED between January 1, 2000, and March 31, 2017. Sildenafil became available in the United Kingdom as an over the counter (OTC) purchase without a prescription from April 2018.^23^ We set the end of exposure ascertainment as March 31, 2017, to avoid misclassifying exposed users who purchased OTC sildenafil as nonexposed. The end of follow-up was set as March 31, 2018, to allow all individuals to have at least 1-year follow-up.

The date of ED diagnosis was defined as cohort entry. We excluded patients with less than 6 months record history before cohort entry. To minimize the inclusion of prevalent users, all patients were required to have never been prescribed PDE5I at any time before cohort entry. We also excluded patients with a recorded diagnosis of any type of dementia or prescription for a symptomatic treatment of dementia at any time before cohort entry (eTables 1 and 2, links.lww.com/WNL/D371). Patients who had a record of cognitive impairment or confusion any time before cohort entry were also excluded to minimize the inclusion of individuals with prodromal dementia. Finally, patients using nicorandil or nitrate-based drugs within 90 days before cohort entry were also excluded because PDE5I are contraindicated in users of these therapies. A graphical depiction illustrating the key aspects of our study design is shown in eFigure 1 (links.lww.com/WNL/D370).

### Exposure

The exposure of interest was initiation of a PDE5I (sildenafil, tadalafil, vardenafil, and avanafil) determined by prescription records, using drug code lists for PDE5I (eTable 3, links.lww.com/WNL/D371). The comparison group was men also newly diagnosed with ED and no prescription for PDE5I (nonusers/nonexposed). The recommended approach for the management of ED in the United Kingdom includes a combination of lifestyle changes, counselling, and drug treatment.^24^

To address the potential for immortal time bias arising from misclassified person-time and to accurately allocate person-time at risk to the exposed and nonexposed groups, measures were taken as outlined in the literature.^25,26^ At cohort entry (ED diagnosis), all patients were categorized as nonexposed and had their person-time reassigned to the exposed group if they were prescribed a PDE5I during the follow-up. Patients who received their first PDE5I prescription on the same date as cohort entry (ED diagnosis) were included in the exposed group from the outset of the study. All subsequent person-time after the initiation of a PDE5I was classified as exposed (eFigure 2, links.lww.com/WNL/D370). Patients diagnosed with ED who were never issued a prescription for a PDE5I remained in the nonexposed group for the entirety of their follow-up.

### Outcome

The primary outcome was the first recording of AD during follow-up, identified using diagnostic read codes linked to its clinical diagnosis (eTable 1, links.lww.com/WNL/D371). Patients were followed-up until the first occurrence of the study outcome, death, transfer out of their general practitioner (GP) practice, the last date of GP data availability or the end of the study period (March 31, 2018), whichever came first.

### Covariates

We adjusted for an extensive number of covariates all measured before or at cohort entry. These included risk factors for AD and potential confounders associated with PDE5I exposure and AD. At cohort entry, we measured age and calendar year—categorized as 2000–2004, 2005–2009, 2010–2013, and 2014–2017 to reflect changes in UK availability of different PDE5Is because of licensing and guidelines.^27^ The model also included the following covariates measured any time before cohort entry, taking the measurement closest to cohort entry date: alcohol status (drinker, ex-drinker, and nondrinker), smoking status (current, ex-smoker, and never), body mass index (BMI) (underweight; 18.59 kg/m^2^, healthy weight; 18.6–24.9 kg/m^2^, overweight; 25–29.9 kg/m^2^, obese; 30–39.9 kg/m^2^, severely obese; ≥40 kg/m^2^), Townsend deprivation quintile score (1; least deprived and 5; most deprived), and all the comorbidities listed in Table 1. We also included the average mean systolic and diastolic blood pressure taken from 12 months before the cohort entry. Finally, recent coprescribed medications (issued on or within 90 days before cohort entry) were also included in the model (Table 1). All covariates were remeasured for individuals who had their person-time reassigned at the point of PDE5I initiation.

**Table 1:** Baseline Characteristics of Patients Included in the Study Cohort Before and After IPTW Using Propensity Scores

	Before IPTW			After IPTW^a^		
	PDE5-inhibitor user (n = 148,338)	Nonuser (n = 121,387)	SMD	PDE5-inhibitor user (n = 147,989)	Nonuser (n = 121,736)	SMD
Age, y, mean (SD)	58 (10)	59 (11)	0.05	58 (10)	58 (10)	0.01
Alcohol status, n (%)			0.06			0.01
Drinker	130,701 (88)	104,532 (86)	—	129,301 (87)	106,126 (87)	—
Ex-drinker	3,589 (2)	3,377 (3)	—	3,777 (3)	3,145 (3)	—
Nondrinker	14,048 (10)	13,478 (11)	—	14,911 (10)	12,465 (10)	—
Smoking status, n (%)			0.01			<0.01
Current	31,731 (21)	26,649 (22)	—	32,138 (22)	26,421 (22)	—
Ex-smoker	50,492 (34)	40,869 (34)	—	49,968 (34)	41,154 (34)	—
Never	66,115 (45)	53,869 (44)	—	65,883 (44)	54,161 (44)	—
BMI, kg/m^2^, n (%)			0.07			<0.01
Underweight (<18.59)	717 (1)	582 (1)	—	712 (0)	585 (0)	—
Healthy weight (18.6–24.9)	36,655 (25)	28,307 (23)	—	35,735 (24)	29,302 (24)	—
Overweight (25–29.9)	66,992 (45)	53,286 (44)	—	66,024 (45)	54,348 (45)	—
Obese (30–39.9)	40,611 (27)	35,582 (29)	—	41,723 (28)	34,345 (28)	—
Severely obese (≥40)	3,363 (2)	3,630 (3)	—	3,795 (3)	3,157 (3)	—
Townsend quintile, n (%)			0.06			0.01
1 (least deprived)	42,417 (29)	32,199 (27)	—	41,138 (28)	33,566 (28)	—
2	34,602 (23)	27,982 (23)	—	34,265 (23)	28,287 (23)	—
3	30,490 (21)	25,291 (21)	—	30,542 (21)	25,161 (21)	—
4	24,503 (16)	21,212 (17)	—	25,039 (17)	20,636 (17)	—
5 (most deprived)	16,326 (11)	14,703 (12)	—	17,006 (11)	14,087 (11)	—
Systolic BP, mm Hg, mean (SD)^b^	138 (15)	138 (15)	0.04	138 (15)	138 (15)	0.01
Diastolic BP, mm Hg, mean (SD)^b^	82 (9)	82 (10)	0.01	82 (9)	82 (9)	<0.01
Comorbidities, n (%)						
Cerebrovascular	5,628 (4)	5,646 (5)	0.04	6,031 (4)	5,082 (4)	<0.01
Heart failure	2,488 (2)	2,952 (2)	0.05	2,851 (2)	2,461 (2)	0.01
Hypertension	86,183 (58)	71,688 (59)	0.02	86,234 (58)	71,113 (58)	<0.01
Ischemic heart disease	22,126 (15)	20.984 (17)	0.06	23,334 (16)	19,454 (16)	0.01
Arrhythmias	5,919 (4)	5,814 (5)	0.04	6,250 (4)	5,285 (4)	0.01
Peripheral vascular disease	2,703 (2)	2,791 (2)	0.03	2,931 (2)	2,472 (2)	<0.01
Dyslipidaemia	22,095 (15)	18,399 (15)	0.01	22,023 (15)	18,160 (15)	<0.01
Diabetes (type 1 and 2)	31,302 (21)	34,868 (29)	0.18	35,652 (24)	29,754 (24)	0.01
Renal disease	7,744 (5)	8,605 (7)	0.08	8,512 (6)	7,291 (6)	0.01
Depression	33,270 (22)	25,949 (21)	0.03	32,518 (22)	26,700 (22)	<0.01
Anxiety	13,743 (9)	10,902 (9)	<0.01	13,540 (9)	11,117 (9)	<0.01
Hypothyroidism	3,466 (2)	3,123 (3)	0.02	3,540 (2)	2,994 (2)	<0.01
Liver disease	3,648 (3)	3,111 (3)	0.01	3,691 (3)	3,030 (3)	<0.01
Benign prostate hyperplasia	11,230 (8)	8,315 (7)	0.03	10,640 (7)	8,771 (7)	<0.01
Schizophrenia/psychosis	992 (1)	1,042 (1)	0.02	1,101 (1)	911 (1)	<0.01
Bipolar disorder	780 (1)	676 (1)	<0.01	796 (1)	656 (1)	<0.01
COPD	4,754 (3)	4,459 (4)	0.03	4,944 (3)	4,124 (3)	<0.01
Head injury or trauma	4,969 (3)	4,013 (3)	<0.01	4,936 (3)	4,051 (3)	<0.01
Coprescribed medications, n (%)^c^						
Lipid-lowering therapy	46,892 (32)	38,774 (32)	0.01	46,481 (31)	38,325 (32)	<0.01
Noninsulin diabetes drugs	21,357 (14)	22,241 (18)	0.11	23,618 (16)	19,586 (16)	<0.01
Insulins	4,740 (3)	4,407 (4)	0.02	5,002 (3)	4,131 (3)	<0.01
Antihypertensives^d^	60,332 (41)	52,353 (43)	0.05	61,455 (42)	50,830 (42)	<0.01
Antidepressants	15,913 (11)	12,071 (10)	0.03	15,345 (11)	12,584 (10)	<0.01
Antipsychotics	1,498 (1)	1,422 (1)	0.02	1,586 (1)	1,306 (1)	<0.01
Anticholinergic drugs^e^	7,933 (5)	6,198 (5)	0.01	7,699 (5)	6,313 (5)	<0.01
Antiplatelets	24,713 (17)	21,507 (18)	0.03	25,094 (17)	20,789 (17)	<0.01
Anticoagulants	3,644 (3)	3,778 (3)	0.04	3,921 (3)	3,337 (3)	0.01
Thyroid replacement therapy	3,422 (2)	2,989 (3)	0.01	3,454 (2)	2,867 (2)	<0.01
Calendar year, n (%)			0.16			<0.01
2000–2004	32,594 (22)	30,744 (25)	—	35,291 (24)	28,907 (24)	—
2005–2009	51,080 (34)	33,411 (28)	—	46,887 (32)	38,548 (32)	—
2010–2013	40,934 (28)	38,806 (32)	—	43,007 (29)	35,601 (29)	—
2014–2017	23,730 (16)	18,426 (15)	—	22,804 (15)	18,680 (15)	—

Abbreviations: BMI = body mass index; BP = blood pressure; COPD = chronic obstructive airways disease; IPTW = inverse probability treatment weighting; SMD = standardized mean difference.

a IPTW was performed after multiple imputation of chained equations.

b Mean systolic and diastolic BP in the 12 months before cohort entry.

c Measured up to 3 months before cohort entry.

d Antihypertensives = angiotensin-converting enzyme inhibitors, angiotensin II receptor antagonists, beta-adrenoreceptor antagonists, calcium channel blockers, diuretics, and a1-adrenoceptor antagonists.

e Drugs with an anticholinergic burden ≥3.

### Statistical Analysis

Descriptive statistics were used to summarize and compare the characteristics of each group at baseline. Categorical variables are reported as counts and percentages, whereas continuous variables are reported using means and SD or medians and interquartile ranges (IQRs). We estimated the crude incidence rate of AD, expressed per 10,000 person-years at risk (PYAR) with 95% CIs.

To address potential confounding bias because of nonrandomization of PDE5I treatment initiation, propensity scores (PSs) were estimated for each patient at baseline, using a logistic regression model conditional on the covariates listed above. The PS is defined as the conditional probability of receiving the treatment of interest (PDE5I) based on a given set of baseline characteristics^28^ (Table 1) and was further used to calculate the inverse probability of treatment weighting (IPTW).^29^ The use of IPTW creates a weighted cohort of patients who differ regarding PDE5I exposure but are similar with respect to the measured characteristics included in the PS model. This method makes it possible to derive estimates representing the average treatment effect in the whole ED population.^29^ To mitigate the possibility of extreme weights and reduce the potential for unmeasured confounding, we used stabilized-IPTW.^29^ Stabilized weights take the marginal probability of exposure in the numerator for IPTW calculations. The final weights were further truncated at the 99th percentile. Standardized mean differences (SMDs) were used to assess balance across all covariates in the exposed and nonexposed groups before and after weighting. An SMD <0.1 was considered negligible imbalance.^30^

Multiple imputation by chained equations was used to impute missing data on systolic and diastolic blood pressure (18% missingness), alcohol status (13% missingness), Townsend deprivation score (10% missingness), smoking status (6% missingness), and BMI (5% missingness).^31^ All covariates listed in Table 1, the outcome as a binary indicator, and the Nelson–Aalen estimate of the cumulative hazard to the survival time were included in the imputation model to derive 20 imputed data sets.^32^ The PS for IPTW was further obtained separately in each imputed data set and combined using Rubin's rules to provide an overall estimate.^33^

For the primary analysis, we fitted a time-dependent Cox proportional hazards model to estimate the adjusted (weighted) hazard ratio (HR) of incident AD, comparing PDE5I initiation and nonuse. The HR obtained from each imputed data set was also combined using Rubin's rules^33^ and robust variance estimation used to calculate the standard errors for 95% CI after weighting.

We conducted several secondary analyses. First, we considered 2 additional outcome definitions. For the first, we used a definition of AD-dementia based on diagnostic read codes linked to both AD and unspecified dementia (excluding vascular and other dementias). In the second, to reduce the likelihood of outcome misclassification, we used a high specificity outcome definition of AD, requiring both a diagnostic read code for AD and ≥1 prescription for symptomatic treatment (donepezil, rivastigmine, galantamine, or memantine). The first date between the 2 was assigned as the outcome date. We also carried out an analysis to explore the association between AD and the total number of PDE5I prescriptions issued. This was performed in a time-dependent manner, by updating person-time in each prespecified prescription category based on the cumulative number of prescriptions issued over the follow-up period. This was to avoid immortal time bias in this type of analysis.^34^ The categories for the number of prescriptions issued were: 1, 2–10, 11–20, 21–50, and >50. The reference category for all analyses was nonuse of PDE5I.

To assess the robustness of our primary result, we conducted 3 sensitivity analyses: (1) 1 and 3 year (post hoc) lag periods where all individuals with less than 1 and 3 years of follow-up from cohort entry (ED diagnosis), including those with AD diagnosis were excluded from analysis, with follow-up for the remaining individuals starting after the predefined lag period. This sensitivity analysis explores the prodromal stage between the neurologic onset of AD and recorded diagnosis. It also removes erroneous associations between PDE5I exposure and AD shortly after drug initiation and addresses the possibility of protopathic bias.^35^ (2) 1-year symptom to diagnosis: to reduce misclassification of disease onset, the outcome date in both groups was assigned 1 year earlier than the recorded date of incident AD diagnosis. In this analysis, the last 1 year of follow-up was also excluded for those who were censored without AD. (3) complete-case analysis: we carried out our analysis without multiple imputation, restricted to individuals with no missingness in measured covariates.

Prespecified subgroup analyses were further conducted, with each adjusted HR estimated separately from recalculated PS and IPTW.^36^ We assessed the association varied by type of PDE5I, categorized into 3 mutually exclusive time-varying exposure groups: sildenafil, tadalafil, and vardenafil. Avanafil was not explored because of the low number of individuals exposed to this type of PDE5I. We also performed subgroup analyses by age (<70 and ≥70 years) to explore whether the association varies within a higher age-specific risk group for AD. In addition, subgroup analyses were performed in individuals with and without a history of hypertension and diabetes, which are both strong risk factors for ED and AD.^37,38^

A *p* value <0.05 was considered statistically significant. All statistical analyses were conducted in Stata version 17 (Stata Statistical Software: release 17, 2021; StataCorp LLC, College Station, TX).

### Standard Protocol Approvals, Registrations, and Patient Consents

This study was approved by the IMRD-UK Scientific Review Committee in March 2022 (Reference Number: 20SRC003-A1).

### Data Availability

Anonymized patient data provided by IMRD were used to conduct this study. The data are anonymous, encrypted, and managed in accordance with General Data Protection Legislation and Ethical regulations in the United Kingdom. Therefore, the data are not widely available for public dissemination. However, applications for access can be made from academic research and pharmaceutical industry entities through direct contact with Cegedim Healthcare Solutions.

## Results

A total of 413,858 men with newly diagnosed ED were identified between January 1, 2000, and March 31, 2017. After applying the exclusion criteria, 269,725 men were included in the study (eFigure 3, links.lww.com/WNL/D370). The mean age at cohort entry was 58.5 (SD = 10.0) years and the median follow-up was 5.1 (IQR 2.9–8.9) years. Baseline characteristics are shown in Table 1 before and after IPTW. All measured covariates were well balanced across the exposed and nonexposed groups after IPTW, with standardized mean differences <0.1.

**Figure 1:**
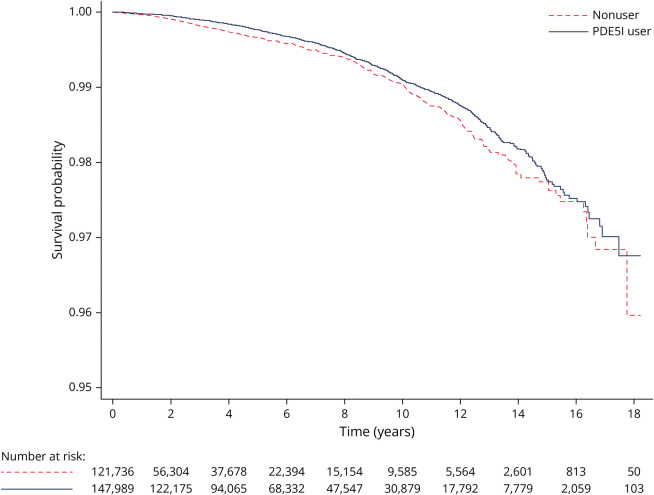
Weighted Kaplan-Meier Curve for the Incidence of Alzheimer Disease During the Follow-Up Period PDE5I = phosphodiesterase type 5 inhibitor.

**Figure 2:**
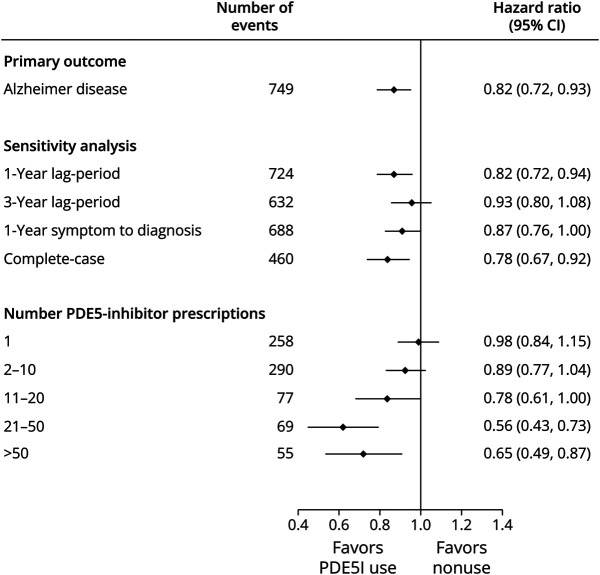
Forest Plot Summarizing the Results of Secondary and Sensitivity Analyses for the Association Between the Use of PDE5I and the Risk of Incident Alzheimer Disease PDE5I = phosphodiesterase type 5 inhibitor.

During 1,309,205 person-years of follow-up, there were 1,119 individuals newly diagnosed with AD. The characteristics of these patients diagnosed with AD is presented in eTable 4 (links.lww.com/WNL/D371). In those exposed to PDE5I, 749 developed AD, corresponding to a crude incident rate (IR) of 8.1 per 10,000 PYAR (95% CI 7.5–8.7). In the nonexposed group, 370 men developed AD, corresponding to a crude IR of 9.7 per 10,000 PYAR (95% CI 8.7–10.7). We found evidence that the initiation of PDE5I was associated with a decreased risk of AD compared with nonusers (adjusted HR 0.82, 95% CI 0.72–0.93) (Table 2). The IPTW-adjusted Kaplan–Meier curve for the incidence of AD is shown in Figure 1. In secondary analyses, we also identified a reduced risk of AD among those exposed to PDE5I when the outcome definition was varied to include a broader and highly specific characterization of AD (Table 2). Furthermore, in an analysis cumulating the total number of PDE5I prescriptions issued, our results show that individuals issued 21–50 or >50 prescriptions were associated with a reduced risk of AD compared with nonusers (adjusted HR 0.56, 95% CI 0.43–0.73 and 0.65 95% CI 0.49–0.87, respectively). However, no evidence of reduced risk was observed in individuals issued with a 1, 2–10 or 11–20 prescriptions compared with nonusers (Figure 2).

**Table 2:** Number of Events, IRs, Unadjusted, and Adjusted HRs for the Primary Analysis and Secondary Outcome Definitions Assessing the Association Between PDE5Is vs Nonuser and Risk of AD in a Cohort of Men With Erectile Dysfunction

Analysis	Events	Person-years	Crude IR (95% CI)^a^	Unadjusted HR (95% CI)	Adjusted HR (95% CI)^b^
Primary: AD					
Nonuser	370	383,236	9.7 (8.7–10.7)	Ref	Ref
PDE5I user	749	925,969	8.1 (7.5–8.7)	0.74 (0.66–0.84)	0.82 (0.72–0.93)
Secondary outcome definitions					
AD + unspecified dementia					
Nonuser	683	382,605	17.9 (16.6–19.2)	Ref	Ref
PDE5I user	1,247	924,669	13.5 (12.8–14.3)	0.68 (0.62–0.74)	0.76 (0.69–0.83)
AD + ≥1 prescription					
Nonuser	258	383,383	6.7 (6.0–7.6)	Ref	Ref
PDE5i user	533	926,279	5.8 (5.3–6.3)	0.76 (0.65–0.88)	0.83 (0.71–0.97)

Abbreviations: AD = Alzheimer disease; HR = hazard ratio; IR = incidence rate; PDE5I = phosphodiesterase type 5 inhibitor.

a Per 10,000 person-years.

b Adjusted estimate represents the results from propensity score-derived stabilized inverse probability of treatment weights: covariates included are age, calendar year, heart failure, chronic obstructive airways disease, dyslipidemia, diabetes, arrhythmias, ischemic/coronary heart disease, cerebrovascular disease, peripheral vascular diseases, renal disease, hypertension, schizophrenia and psychosis, bipolar disorder, depression, anxiety, liver disease, benign prostate hyperplasia, hypothyroidism, head injury/trauma, Townsend deprivation quintile, mean systolic and diastolic blood pressure, body mass index, smoking status, alcohol status, antidepressants, noninsulin diabetes treatments, insulins, antipsychotics, antihypertensives, antiplatelets, anticoagulants, anticholinergic drugs with an anticholinergic burden ≥3, lipid-lowering therapies, and thyroid replacement therapies.

**Figure 3:**
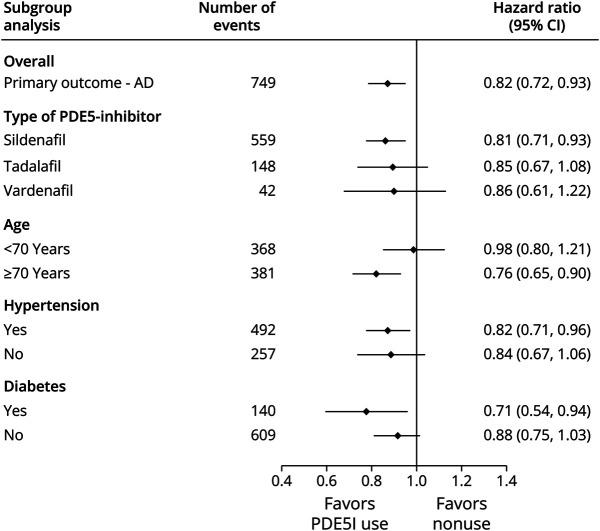
Forest Plot Summarizing the Results of Subgroup Analyses for the Association Between the Use of PDE5I and the Risk of Incident AD AD = Alzheimer disease; PDE5I = phosphodiesterase type 5 inhibitor.

The primary result was consistent with sensitivity analyses that introduced a 1-year lag (delay) period from cohort entry, 1-year symptom to diagnosis period, and inclusion of complete cases only (Figure 2). However, in an analysis extending the lag period to 3 years, our findings were not consistent with the primary result (HR 0.93, 95% CI 0.80–1.08). In subgroup analyses, we identified evidence of reduced AD risk in those initiated on sildenafil (adjusted HR 0.81, 95% CI 0.71–0.93). The effect estimates for those initiated on tadalafil and vardenafil were similar, but we did not find strong evidence for reduced risk compared with nonusers. The CIs for these 2 exposure subgroups were relatively wide because of the smaller number of individuals exposed and cases of AD (Figure 3). In other subgroup analyses, lower risk of AD in PDE5I users compared with nonusers was observed across strata of patients with hypertension, diabetes, and in older men aged ≥70 years. We did not find strong evidence to suggest an association in men <70 years or those without a background of hypertension or diabetes.

## Discussion

In this large population-based cohort study of men in the United Kingdom aged ≥40 years with ED, we found that compared with nonuse, those who were exposed to PDE5I had a reduced risk of developing incident AD. Individuals issued with a greater number of PDE5I prescriptions demonstrated more benefit compared with nonusers. The results were robust to secondary analyses exploring different outcome definitions, but not in all sensitivity analyses that incorporated different analytical methods. Specifically, inclusion of a 3-year lag period did not provide strong evidence of association. There is a latent period between AD onset and diagnosis. The optimal period is unknown; therefore, we sought to explore this by including a 1- and 3-year lag period in our sensitivity analysis. As the lag period was extended, the strength of evidence for reduced risk associated with PDE5I exposure weakened. This uncertainty in our sensitivity analysis may reflect the impact of latency bias on our primary result. However, it is also important to acknowledge that the application of an increasing lag period can lead to a decrease in statistical power. This decrease is attributed to the loss in sample size, resulting in reduced precision, and is reflected by wider CIs.

Across different subgroups, we reported a reduced risk of AD in those exposed to sildenafil but not tadalafil or vardenafil. PDE5I exposure in older aged men (≥70 years) and in individuals with a history of hypertension and diabetes were also associated with a reduced risk from AD. However, this association varied across different subgroups within the ED population. Among individuals without hypertension or diabetes and those <70 years, we did not find evidence of reduced AD risk from PDE5I exposure. Although this analysis is exploratory and performed in a smaller subset of patients, these findings cautiously allude to greater benefit from PDE5I use in individuals at greatest risk of AD and warrant further study to investigate this hypothesis.

Evidence from pharmacokinetic studies have shown that the PDE5Is, sildenafil and tadalafil, both cross the blood–brain barrier, with sildenafil demonstrating greater permeability.^39,40^ Therefore, the availability of these drugs in the CNS where they can exert their inhibitory effect on expressed PDE in brain cells may be suggestive of the mechanism that contributes to some level of neuroprotection.^11,12^ The protective effect we observed from the increasing cumulative number of prescriptions may also suggest that repeated exposure can lead to greater accumulation of PDE5I in the brain of humans as observed in animal models in which neurologic benefit after chronic PDE5I exposure was demonstrated.^40^

The association between PDE5I and incident AD has been assessed by 2 previous observational studies. In keeping with findings by Fang et al.,^16^ we identified a reduced risk of AD in our primary analysis but not to the scale of 69% that was observed in their study. We restricted our cohort to men with ED, therefore using a homogenous population and included a more extensive list of covariates in our PS-model to adjust for baseline confounding and minimize selection bias. We also treated exposure to PDE5I as a time-varying variable, therefore ensuring person-time at risk was correctly allocated to minimize immortal-time bias. Work by Desai et al.^17^ found no association between PDE5I initiation and AD. However, it is difficult to correctly draw comparisons between our work because their new-user active comparator study was carried out in a different target population (pulmonary arterial hypertension) and compared PDE5I initiation with endothelin receptor antagonists in a cohort with 69% female sex. The benefit of the UK database we used made it possible to include additional key variables in our analysis, such as blood pressure, alcohol status, BMI, and socioeconomic status, which is commonly unavailable in US insurance claims data. However, the analyses by Desai et al. did include possible markers of behavior and frailty such as colonoscopy and mammography, and therefore, we cannot rule out an underlying level of unmeasured confounding in our results. Nevertheless, in their analysis akin to intention-to treat, the median follow-up was under 2 years for less than 5,000 patients compared with 5.1 years in our study that included 269,725 patients. The methods implemented by Desai et al. were rigorous, but it is possible their study was underpowered to detect differences of small magnitude and the length of follow-up too short to estimate effects of longer duration.

This study has several limitations. First, PDE5I exposure was based on prescription records, and therefore, we do not know if patients collected or used the prescribed treatment. Misclassification of PDE5I exposure may have occurred to some extent. However, PDE5I for ED are prescribed on an “as needed” basis and are most likely to be used by those motivated to take their medication for ED. Any bias resulting from misclassification is likely to be nondifferential regarding AD and dilute any measured effect toward the null. Second, we did not have information from diagnostic brain imaging and autopsy to confirm the accuracy of AD diagnosis based on clinical read codes. However, diagnosis of dementia in primary care, including AD, has a reported specificity of 83% and minimal false negatives in a sample without recorded dementia.^41^ Moreover, to address possible outcome misclassification, we repeated the primary analysis using different outcome definitions that gave results consistent with the main findings. Third, we could not accurately assess a dose-response relationship because the duration of PDE5I treatment is not adequately recorded, given its “as needed” frequency of use for management of ED. To mitigate, we conducted all analyses assuming individuals remained exposed after PDE5I initiation, regardless of subsequent discontinuation or treatment changes, similar to an intention-to-treat approach in randomized clinical trials. This assumption also addresses concerns related to informative censoring. In our study, this could be apparent where patients discontinue PDE5I by choice or are deprescribed because of unrecorded memory problems associated with AD. We also for the first time investigated the effect from the cumulative number of prescriptions issued over the follow-up period. The results from this secondary analysis can be cautiously interpreted as a proxy for the regularity of use. However, we cannot rule out graded confounding in line with cumulative prescriptions. For example, the higher number of prescriptions may be correlated with risk factors for ED (e.g., uncontrolled diabetes) that also confound the association with AD. Nevertheless, we used PSs to calculate IPTW and balance the distribution of confounders across comparator groups. Last, because of the limitations of our database, we cannot rule out the effect of unmeasured confounding. We were unable to account for levels of physical and sexual activity, which may confer a level of benefit in AD risk and be predictive for PDE5I exposure. We were also unable to adjust for ethnicity because of the high degree of missingness in our study population (66% missing). Previous work by Mukadam et al.^42^ reported variations in the incidence of dementia among different ethnic groups in England. Therefore, a level of residual confounding may affect our results. Nonetheless, we attempted to mitigate this limitation by adjusting for the known dementia risk factors that may disproportionately affect certain ethnic groups, for example, hypertension, diabetes, and obesity.^42^

The findings of this large population-based study suggest that the use of PDE5I may be associated with a reduced risk of incident AD. The greatest risk reduction was observed in those issued >20 prescriptions over a median follow-up of 5 years. This study warrants further investigation into the pathophysiologic action of PDE5I and neuroprotection. Further research is also needed to explore the optimal duration of the lag period, which is necessary to adequately address the prodromal phase of AD. To confirm the findings of our results in a wider population, a randomized controlled trial would be beneficial to evaluate the impact of PDE5I on AD. Such a study would include male and female participants, without cognitive impairment, randomized to receive a PDE5I in predefined doses or placebo. The primary outcomes would be the change in baseline cognitive function. This approach would provide a comprehensive understanding of the potential therapeutic benefits of PDE5I and AD.

## Appendix Authors

**Table:**

Name	Location	Contributions
Matthew Adesuyan, MPharm	Research Department of Practice and Policy, UCL School of Pharmacy; Centre for Medicines Optimisation Research and Education, University College London Hospitals NHS Foundation Trust, London, United Kingdom	Drafting/revision of the manuscript for content, including medical writing for content; major role in the acquisition of data; study concept or design; and analysis or interpretation of data
Yogini H. Jani, PhD	Research Department of Practice and Policy, UCL School of Pharmacy; Centre for Medicines Optimisation Research and Education, University College London Hospitals NHS Foundation Trust, London, United Kingdom	Drafting/revision of the manuscript for content, including medical writing for content
Dana Alsugeir, PharmD	Research Department of Practice and Policy, UCL School of Pharmacy, London, United Kingdom; Pharmacy Practice Department, College of Clinical Pharmacy, Imam Abdulrahman Bin Faisal University, Dammam, Saudi Arabia	Drafting/revision of the manuscript for content, including medical writing for content, and analysis or interpretation of data
Robert Howard, MD	Division of Psychiatry, University College London, United Kingdom	Drafting/revision of the manuscript for content, including medical writing for content, and analysis or interpretation of data
Chengsheng Ju, MPharm	Research Department of Practice and Policy, UCL School of Pharmacy, London, United Kingdom	Drafting/revision of the manuscript for content, including medical writing for content, and study concept or design
Li Wei, PhD	Research Department of Practice and Policy, UCL School of Pharmacy, London, United Kingdom	Drafting/revision of the manuscript for content, including medical writing for content; study concept or design; and analysis or interpretation of data
Ruth Brauer, PhD	Research Department of Practice and Policy, UCL School of Pharmacy, London, United Kingdom	Drafting/revision of the manuscript for content, including medical writing for content; study concept or design; and analysis or interpretation of data

## Glossary

AD: Alzheimer disease
BMI: body mass index
cGMP: cyclic guanosine monophosphate
ED: erectile dysfunction
GP: general practitioner
IMRD: IQVIA Medical Research Data
IPTW: inverse probability treatment weighting
IQR: interquartile range
IR: incident rate
OTC: over the counter
PAH: pulmonary arterial hypertension
PDE: phosphodiesterase enzyme
PDE5I: phosphodiesterase type 5 inhibitor
PS: propensity score
PYAR: person-years at risk
SMD: standardized mean difference
THIN: The Health Improvement Network
